# Ten Simple Rules for Protecting Research Integrity

**DOI:** 10.1371/journal.pcbi.1004388

**Published:** 2015-10-01

**Authors:** David M. Shaw, Thomas C. Erren

**Affiliations:** 1 Institute for Biomedical Ethics, University of Basel, Basel, Switzerland; 2 Institute and Policlinic for Occupational Medicine, Environmental Medicine and Prevention Research, University Hospital of Cologne, Cologne, Germany; Accelrys, UNITED STATES

Research integrity is frequently highlighted as an essential component of modern medicine and science. Adherence to the ethical principles of one’s profession might seem like a simple task, but research misconduct remains a serious problem. Despite repeated calls for increased emphasis on the importance of research integrity [1–6] and a proliferation of guidelines regulating scientific misconduct at the international, national, and institutional levels [7,8], recent scandals concerning falsification and suppression of results [9,10] suggest that we need to more carefully nurture the ethical integrity of our research endeavours.

Most of the recent controversy concerning scientific misconduct has focused on plagiarism and fabrication of results. This type of malpractice has rightly been universally recognized as a very serious breach of research integrity. However, the focus on these abuses has distracted attention away from other practices which, while they may not jeopardize the scientific process to the same extent, are nonetheless clearly breaches of scientific integrity. Much more could be done to combat authorship misattribution, failure to declare relevant conflicts of interest (COIs), failure to report results, and misuse of metrics in funding decisions—in terms of both establishing stronger guidelines and ensuring their enforcement.

Although many institutions have official integrity guidelines, these frequently act as a window dressing—claiming to address key integrity issues while allowing corrosive low-level misconduct to proliferate. For example, putting pressure on junior researchers to allow guest authors on papers is not only unethical but also makes it more likely that they will see such behaviour as acceptable. Here, we suggest ten simple rules that should be put into action by all research institutions and to which all researchers should adhere in order to ensure ethical behaviour in science and medicine. In our view, all integrity guidelines should mandate these minimum requirements. Rules 1–5 are specific recommendations for particular integrity issues, and Rules 6–10 concern what institutions themselves should do.

## Rule 1: All Papers Submitted Must Contain Contributorship Statements

One of the most well-known integrity-related issues is authorship. Everyone in academia has heard of “guest” [11] and “ghost” [12] authors, and most integrity guidelines now mandate adherence to international guidelines and forbid dishonest authorship attribution [13]. This is fine in principle but insufficient in practice; institutional authorship guidelines often differ from those of journals, and even widely accepted guidelines contain contradictions (for example, the International Committee of Medical Journal Editors [ICMJE] criteria offer two different definitions of authorship [14]). In fact, the existence of authorship and research integrity guidelines may provide a false image of respectability for academia [14], as the numerous conflicts of interest that afflict research institutions make it very difficult for both junior and senior researchers to adhere to such guidelines [15]. For example, if a researcher suspects misattribution of authorship, it is his duty to raise concerns, but this poses a clear COI: if he does, he might risk his job or even his career.

What else can be done to ensure honest authorship attribution? The bar must be raised: integrity guidelines should mandate contributorship statements, which are widely recognized as a much more transparent means of attributing authorship and also have the advantage of avoiding ambiguity about which of several conflicting guidelines are being followed [16]. While it is relatively easy (and erroneously recommended in a reputable journal like *Nature* [11,17,18]) for researchers to add their department head as an author when he didn’t contribute anything to the paper, or even to lie on the declaration form and say that he did in fact contribute, it is much more difficult to actively lie on a contributorship statement. The concept of using statements to make contributors accountable was proposed almost 20 years ago [12]; some journals (including the *Journal of the American Medical Association* [*JAMA*] and the *British Medical Journal* [*BMJ*]) do require contributorship statements, and the ICMJE guidelines recommend their use as well [19]. However, these statements are still not mandated by any major integrity guideline, meaning that researchers are unlikely to be sanctioned by their institutions for failing to state contributions accurately. This is a typical example of paying lip service to research integrity without actually speaking to the problem.

In order to increase transparency, all authors should put contributorship statements in every paper they submit, regardless of journal policies. Journals that refuse to print such statements should be considered blacklisted by ethical researchers. Given that ghost and guest authorship are in a similar category as plagiarism, it is surprising that they are not treated with the same gravity. Ideally, contributorship statements would replace traditional author lists at the start of articles, rather than being buried at the end of the paper, which still allows assumptions about author order to play a role. Such statements are important not only in order to acknowledge non-author contributions but also to make it clear exactly who should take the credit and who bears the responsibility for different aspects of a research paper, rather than letting readers guess based on the order of authors.

Some studies have already investigated whether contributorship statements impart relevant information [20], but we need more empirical evidence regarding how researchers are obeying the many available rules.

## Rule 2: All Financial COIs Must Be Reported with No Time Limits

As well as being honest in terms of authorship, researchers are expected to declare all conflicts of interest when applying for funding, publishing papers, or peer reviewing. Nonetheless, many COI policies and COI clauses in integrity guidelines are weak; even supposedly “comprehensive” checklists can omit items such as previous personal fees paid by sponsors [21]. Requiring a declaration of current financial COIs or only those from the last few years is insufficient when research has shown that even receiving free pens can influence physician decision-making [22]. All financial COIs that could ever have affected a researcher’s judgement should be declared, with no time limits.

## Rule 3: Relevant, Non-financial Potential COIs Must Be Declared

Furthermore, more emphasis should be put on non-financial COI, which can bias researchers just as much as money [23,24]. *The Lancet* has perhaps the best COI policy of any medical journal, even though it limits financial conflicts to only the last three years [25]. It mentions “personal relationships or rivalries, academic competition, or intellectual beliefs” and states that “the editor may use such information as a basis for editorial decisions, and will publish such disclosures if they are believed to be important to readers in judging the manuscript.” This grants great discretion to the editors of the journal, who could themselves be subject to bias when assessing COI.

Let’s face it: anyone who conducts scientific work can, and most likely will, be biased in some way or another. Authors who feel tempted not to disclose competing interests should be clear about the fact that readers continue to act as “peer reviewers” after publication, increasing peer review by orders of magnitude [26]. The penalties for non-disclosure of relevant conflicts must also be severe (see below).

## Rule 4: Trials Must Be Reported Accurately, As Well As Registered

Another important issue concerns clinical trials. While some countries now mandate registration of clinical trials on public registries like clinicaltrials.gov, there are still many that do not, and even those that do still allow exceptions for certain circumstances and first-in-human (FIH) trials. If research integrity is to be taken seriously, there should be no exceptions. Furthermore, despite the emphasis on registration, reporting of results is actually much more important, and registration is only a means to this end [27]. As such, requiring registration without requiring reporting is pointless. Sanctions must be introduced for those not publishing results or publishing only partial results. The United Kingdom National Health Service (NHS) Research Ethics Service recently made trial registration a condition of approval [28], and the European Union’s new clinical trial regulation mandates summaries of results on a new trial registry [29]. These are important steps in the right direction, but full and accurate disclosure of results is necessary to ensure research integrity. Commendably, the Public Library of Science (PLOS) journals now require publication of raw data alongside research articles and adherence to reporting guidelines like CONSORT [30]. Further investment of resources in ethics committees must be sufficient to ensure monitoring of registration and publication of results, and all new projects should only be approved subject to agreements concerning publication and analysis. For example, the Avon Longitudinal Study of Parents and Children requires all projects to guarantee “the right to check that all objectives in the original proposal are completed by cross reference to publications and make any additional analyses that were in the initial proposal but that have not been published via letters to journals and/or on our website, in order to avoid publication bias [31].”

## Rule 5: Any Use of Metrics in Research Decisions Must Be Evidence Based

Another area of research integrity that is often neglected concerns funding and employment decisions. Misuse of impact factors and other metrics means that funding and employment are based on irrelevant measures, which compromises the research process [15]. Any metric-based decisions must be demonstrably evidence-based, possibly with an emphasis on the personal citations of researchers, rather than on impact factors of journals. Misallocation of research funding and misidentification of research priorities can mean that the wrong questions are being asked and the wrong people are being employed as researchers, which also threatens the integrity of the research process [32].

## Rule 6: All Breaches of Integrity Guidelines Should Be Punished or Remediated

An indicator of the way in which low-level misconduct is tolerated is the lack of sanctions for those who breach guidelines. It is hardly surprising that there is little punishment when there is very little detection or enforcement of things like dishonest authorship attribution. In order to change this, palpable sanctions must be introduced. For example, if a journal discovers that a COI was not disclosed by an author, the paper in question could be withdrawn in the same way as if data distortions had been discovered [33,34]. If a researcher is found to have added guest authorship in exchange for some favour, he or she should face disciplinary proceedings without exception. And if a researcher is found to have suppressed or manipulated results, the result of any such proceedings should be dismissal. (In the case of the pharmaceutical industry, in which unethical manipulation of data has been demonstrated, fines for misconduct should be vastly increased.) The financial costs of properly enforcing research integrity policies may be quite high [35], but the moral and scientific cost of failing to act would be greater. The “pyramid of sanctions” approach, with escalating levels of penalties, is likely to be appropriate here [36].

## Rule 7: All Institutions Must Have Clear Procedures for Raising Concerns and Protections for Those Who Do So

A lack of rigor is evident throughout current integrity systems. Assuming that they are aware of the principles of research integrity (see Rule 10), junior researchers, in particular, often have no clear path by which to raise concerns. If such a pathway exists, they often risk losing their jobs or their careers because no protections exist for whistleblowers; this will make many decide not to raise concerns. Even if they are brave enough to do so, there will probably not be defined sanctions that can be brought against the offender. And even if there are, research integrity officers (RIOs) often wield very little power, so there may not be any consequences for the unethical senior researcher, even if there are negative consequences for the ethical junior researcher. All universities and research institutions must have clear pathways for raising concerns, protections for whistleblowers, defined punishments for wrongdoers, and strong powers for RIOs.

## Rule 8: Raising Concerns about Suspected Misconduct Must Be Mandatory

All integrity policies should mandate whistleblowing on integrity issues. To facilitate this, all institutions should provide career protection for those who raise concerns about their superiors. Only if whistleblowers are truly protected will research integrity be fostered.

## Rule 9: RIOs Must Have the Power to Enforce Integrity Policies

Even if integrity guidelines are strengthened in line with these suggestions, strong rules are meaningless without the means to enforce them. As well as making integrity policies stronger and more visible, RIOs at universities, companies, and other research institutions must be given the muscle required to punish those in breach. In practice, this means that RIOs must have the power to initiate academic misconduct proceedings themselves, without explicit approval of deans or others in the university hierarchy.

## Rule 10: Integrity Policies Must Be Highly Visible and Understood

We have assumed so far that researchers are aware both of the basic principles of research integrity and of any relevant institutional or national guidelines. While most researchers probably do have some idea of what constitutes misconduct, very many are unaware of their university’s specific policies [37]. Students and staff must be made aware of the standards they are expected to meet; there is no point in having a zero-tolerance approach to scientific misconduct if no one knows that the standard of research integrity has been set so high. As well as making their own integrity guidelines highly visible, institutions should make integrity education a key part of undergraduate and postgraduate courses across all disciplines.

Clearly, our list of rules is not exhaustive, and other requirements may need to be added. Some will object, claiming that it is not necessary to set the bar so high. However, many people will always do the minimum required in terms of integrity. Therefore, the minimum must be set very high in order to protect research integrity. Some will also argue that it is impractical to set the bar so high for such “small” breaches. But if integrity is truly important, researchers must be vigilant for any and all cases of misconduct, not just the really obvious, really bad cases. The various national and international guidelines [38] must be more specific and better enforced in order to prevent them from being used as a camouflage for misconduct.
